# Diffusion imaging could aid to differentiate between glioma progression and treatment-related abnormalities: a meta-analysis

**DOI:** 10.1186/s13244-022-01295-4

**Published:** 2022-10-04

**Authors:** Rik van den Elshout, Tom W. J. Scheenen, Chantal M. L. Driessen, Robert J. Smeenk, Frederick J. A. Meijer, Dylan Henssen

**Affiliations:** 1 Department of Medical Imaging, Radboud University Medical Center, Geert Grooteplein Zuid 10, 6525 EZ Nijmegen, The Netherlands; 2 Department of Medical Oncology, Radboud University Medical Center, Nijmegen, The Netherlands; 3 Department of Radiation Oncology, Radboud University Medical Center, Nijmegen, The Netherlands

**Keywords:** Apparent diffusion coefficient, Fractional anisotropy, Glioblastoma, Tumor progression, Treatment-related abnormalities

## Abstract

**Background:**

In a considerable subgroup of glioma patients treated with (chemo) radiation new lesions develop either representing tumor progression (TP) or treatment-related abnormalities (TRA). Quantitative diffusion imaging metrics such as the Apparent Diffusion Coefficient (ADC) and Fractional Anisotropy (FA) have been reported as potential metrics to noninvasively differentiate between these two phenomena. Variability in performance scores of these metrics and absence of a critical overview of the literature contribute to the lack of clinical implementation. This meta-analysis therefore critically reviewed the literature and meta-analyzed the performance scores.

**Methods:**

Systematic searching was carried out in PubMed, EMBASE and The Cochrane Library. Using predefined criteria, papers were reviewed. Diagnostic accuracy values of suitable papers were meta-analyzed quantitatively.

**Results:**

Of 1252 identified papers, 10 ADC papers, totaling 414 patients, and 4 FA papers, with 154 patients were eligible for meta-analysis. Mean ADC values of the patients in the TP/TRA groups were 1.13 × 10^−3^mm^2^/s (95% CI 0.912 × 10^–3^–1.32 × 10^−3^mm^2^/s) and 1.38 × 10^−3^mm^2^/s (95% CI 1.33 × 10^–3^–1.45 × 10^−3^mm^2^/s, respectively. Mean FA values of TP/TRA was 0.19 (95% CI 0.189–0.194) and 0.14 (95% CI 0.137–0.143) respectively. A significant mean difference between ADC and FA values in TP versus TRA was observed (*p* = 0.005).

**Conclusions:**

Quantitative ADC and FA values could be useful for distinguishing TP from TRA on a meta-level. Further studies using serial imaging of individual patients are warranted to determine the role of diffusion imaging in glioma patients.

## Key points

- ADC and FA values can differentiate between TP versus TRA.
- ADC is reduced in TP due to reduced extracellular water diffusion
- FA is hypothesized to be reduced in TRA due to necrosis
- Lack of reporting according to FAIR principles lead to excluded papers

## Background

In approximately 40% of high-grade glioma patients (WHO 3 + 4), the combination of chemotherapy and radiation provokes increased contrast agent uptake and apparent enlargement of residual tumor, or the appearance of new lesions mimicking glioma progression, which can also occur as radiation-induced injury in the treatment of low-grade glioma [1–3]. These phenomenon are called Treatment-Related Abnormalities (TRA), its pathophysiology is not known entirely, but it is likely to relate to endothelial cell injury as a consequence of treatment, leading to tissue inflammation and increased edema due to upregulation of VEGF. TRA are described in the literature as pseudoprogression and radio necrosis. It is a benign occurrence which regresses without additional treatment and is related to better outcomes [2]. On conventional magnetic resonance imaging (MRI), transient increases in post-contrast ring-enhancing lesions and increases in surrounding T2/FLAIR hyperintense regions similar to true tumor progression (TP) can be observed [4]. Although conventional MRI can serve to detect changes in the imaging follow-up of glioma, it is not sufficient by itself to distinguish TRA from TP. Therefore, this has become one of the major challenges in the radiological follow-up of glioma patients as only tissue biopsy or serial imaging can provide a conclusive diagnosis [5]. As conventional MRI protocols provide insufficient diagnostic accuracy to distinguish TRA from TP more sophisticated imaging protocols have been suggested. Such imaging protocols include the use of diffusion-weighted imaging (DWI), perfusion-weighted imaging (PWI) and diffusion tensor imaging (DTI). One of the DWI metrics concerns the apparent diffusion coefficient (ADC) of tissue [6]. Processes which degrade cellular integrity (e.g., necrosis) result in increased free water in the extracellular space, increasing ADC value, whereas increased cellularity with tumors reduces extracellular water, and is associated with a lower ADC value [7]. In the clinical setting the ADC value was found to be suitable to assess glioma response to therapy and to predict survival serially, with the same scanner and protocol [8]. In contrast, the DTI metric fractional anisotropy (FA) increases when water is more restricted in its diffusion directions [9]. A higher FA value indicates increased cellularity and reduced isotropy and has been shown to be able to assess glioma infiltration as well [10]. Additionally, various reports have been published with regard to the use of the ADC and/or FA values to distinguish TRA from TP [11, 12]. However, these studies reported different sensitivity/specificity-rates, positive predictive values and threshold-ratios and a clear overview of quantitative ADC or FA measures on a meta-level remains elusive. The present study reviews the evidence on DWI and DTI in the discernment of TRA from TP and assessed whether there is sufficient evidence on a meta-level to support the use of the ADC or FA values in the daily clinical setting. Finally, challenges and opportunities for future clinical studies are elucidated.

## Materials and methods

### Search strategy and inclusion criteria/exclusion criteria

The updated Preferred Reporting Items for Systematic Reviews and Meta-Analyses (PRISMA 2020) guidelines [13] were followed during the conduction of this study. PubMed, EMBASE and The Cochrane Library were searched systematically to retrieve relevant literature published before January 2021. Cross-referencing was used to add relevant literature to the database. Searches were conducted between May 1, 2020 and January 1, 2021.

In order to be eligible for this review, studies needed to describe the use of the ADC or FA values to distinguish TRA from TP in post-treatment glioma patients. To be included (1) studies needed to provide statistics with regard to the ADC/FA values or these data could be retrieved by contacting the corresponding author; (2) ADC/FA data needed to be presented for the TRA and TP group separately; (3) grading of the included glioma needed to be presented following the World Health Organization (WHO) grading system; (4) demographics of the included patients needed to be presented for each group separately (e.g., sex, mean age) and (5) information on the MR imaging protocol, especially the used b-value needed to be reported as b-values, among others, impact the diffusion metrics [14]. Papers were excluded if they were letters, preprints, case reports, congress proceedings, narrative reviews or when based on animals or pediatric populations.

Retrieved papers were assessed in three rounds by two researchers independently (an MD-PhD student with 2  years of research experience and a fourth-year resident in radiology/nuclear medicine holding a PhD in neuro-imaging with over 7 years of expertise in experimental imaging of the brain). The first round comprised screening on title and abstract; the second round comprised full-text analysis to assess whether the papers met any inclusion criteria and/or exclusion criteria. In the third round, information was extracted from the included papers.

Data extracted from each study were (a) first author and year of publication, (b) number of patients included in the TRA and TP groups, (c) mean age of the included participants (per group), (d) sex of the included participants (per group), (e) WHO grade of the glioma, (f) ADC/FA metrics (e.g., mean values, standard deviation, median values, quartiles, ranges), (g) sensitivity/specificity rates and (h) MR imaging protocol (e.g., *b*-values). Researchers met periodically to discuss their findings, cross-check data and resolve discrepancies.

### Meta-analysis

Meta-analysis of the yielded quantitative data was carried out using Review Manager (RevMan) (*IOS Version 5.3. Copenhagen: The Nordic Cochrane Centre, The Cochrane Collaboration, 2014*) and OpenMetaAnalyst (*IOS Version 12.11.14. MetaAnalyst, Tufts Medical Center*). A random-effects model was used to compute mean differences in ADC and FA values across studies. Additionally, to assess sensitivity and specificity of the ADC value to distinguish TRA from TP, a diagnostic test accuracy review using a single test approach was conducted. Furthermore, pooled sensitivity/specificity ratio for the included studies were assessed. Heterogeneity was assessed by use of the *I*^2^ statistic; *I*^2^ ranges from 0% (i.e., no heterogeneity) to 100% (i.e., the highest heterogeneity). To calculate *I*^2^ in order to assess its impact on the analysis, first the Chi^2^-value and degrees of freedom were calculated. Moreover, the estimated standard deviation of the distribution of true effect size (Tau^2^) was calculated to assign weights to the studies for the random-effects model. The test for overall effect using the Z-test was also calculated to examine whether the pooled estimate of effect is statistically significant. To evaluate the risk of bias and applicability of primary diagnostic accuracy studies, the QUADAS-2 checklist was used. To determine publication bias, the Egger’s regression test was used.

## Results

**Fig. 1:**
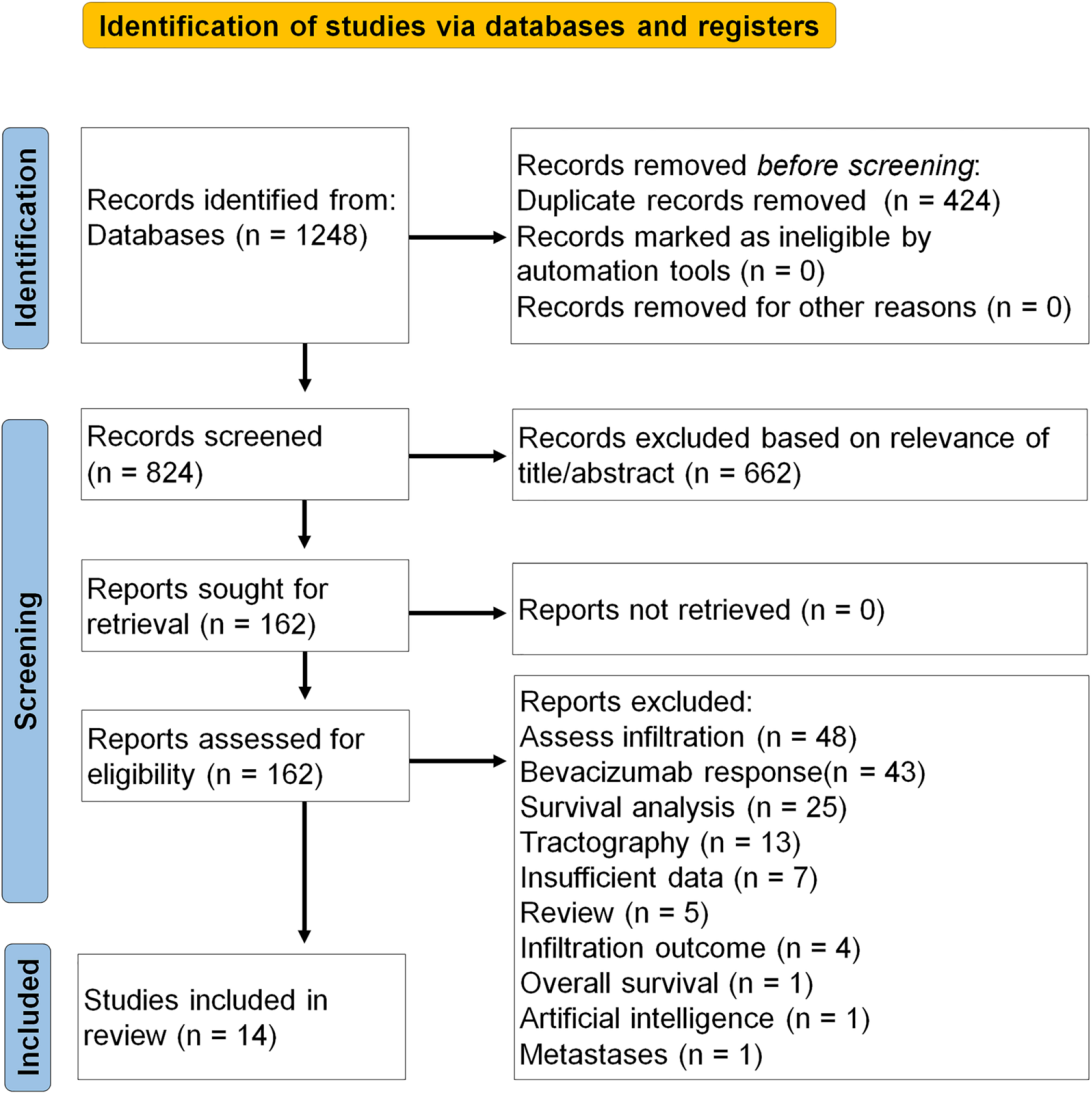
PRISMA 2020 Flow Diagram

**Fig. 2:**
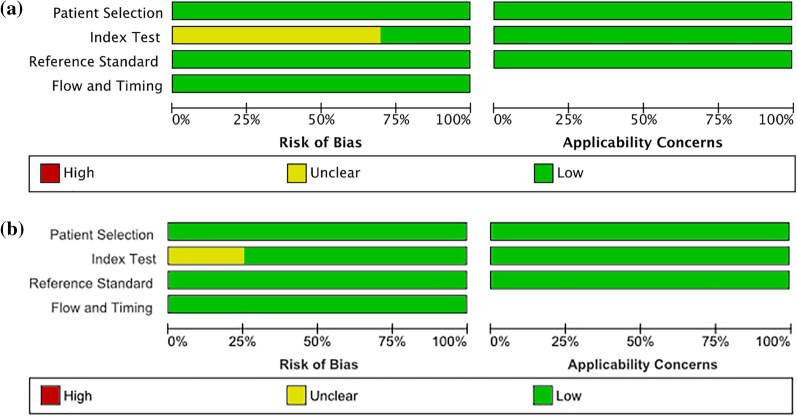
**a** Barplot of risk of bias and applicability concerns across all ADC studies assessed by QUADAS2. **b** Barplot of risk of bias and applicability concerns across all FA studies assessed by QUADAS2

A total of 820 unique studies were identified by systematic searching; four papers were retrieved through cross-referencing. The papers (*n* = 824) were systematically screened on title and abstract. Based on title and abstract, 662 papers were deemed irrelevant and therefore excluded. The 162 remaining papers were included for full-text analysis. In 52 papers, the primary goal of the study was to assess the infiltration of the glioma in the surrounding brain parenchyma, whereas in 43 papers, response prediction to bevacizumab was the primary outcome. Twenty-six studies focused on the use of diffusion metrics in survival analysis. In thirteen papers, the authors focused on the use of tractography. Seven papers provided insufficient data to be included. Five reviews were excluded; one paper concerned a study on brain metastases and one paper focused on implementing artificial intelligence to discriminate TP from TRA. Ten papers using ADC measurements were included in the meta-analysis [11, 12, 15–22]. Four papers using FA measurements were included for analysis [12, 16, 23, 24]. The WHO classification used to differentiate between tumor grade differs between papers depending on the date of publication. Both WHO 2007 criteria and WHO 2016 criteria have been reported. The differentiation in definite diagnosis between TRA and TP was made using either histopathologic findings after a second look operation [12] or by using the Response Assessment in Neuro-Oncology (RANO) criteria, based on radiological and clinical follow-up over time (i.e., newly appearing enhancing lesion outside of the radiation field or clinical confirmation of disease progression [25]). For the PRISMA flowchart, please see Fig. 1. As assessed by the QUADAS-2 tool, the risk of bias was considered low in all included studies (Fig. 2a, b).

### Meta-analysis on the use of apparent diffusion coefficient

**Fig. 3:**
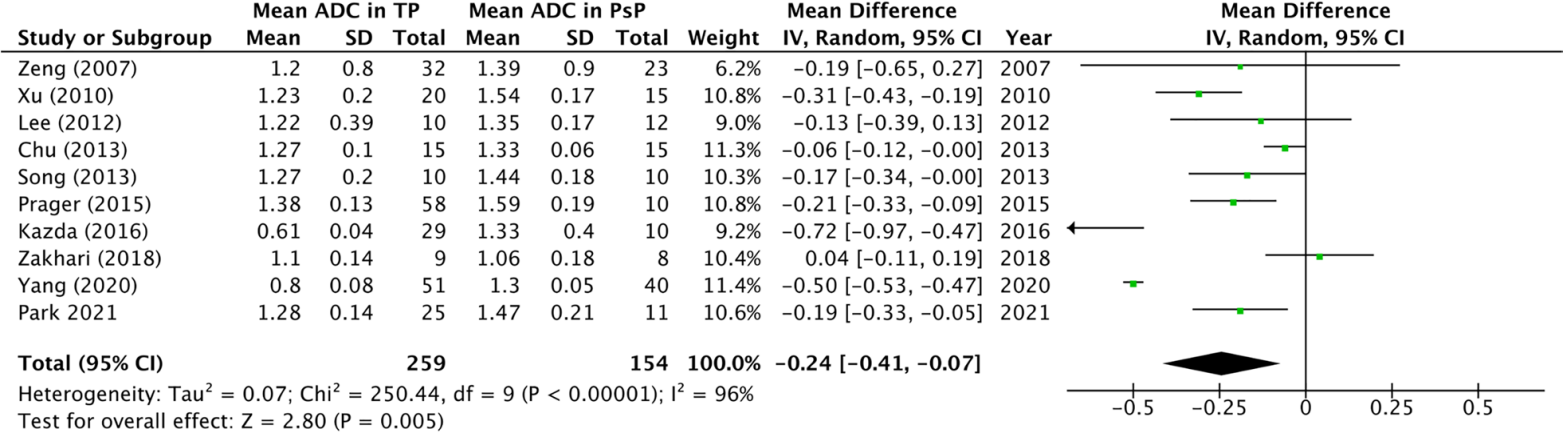
Forest plot of included studies on ADC metrics

**Fig. 4:**
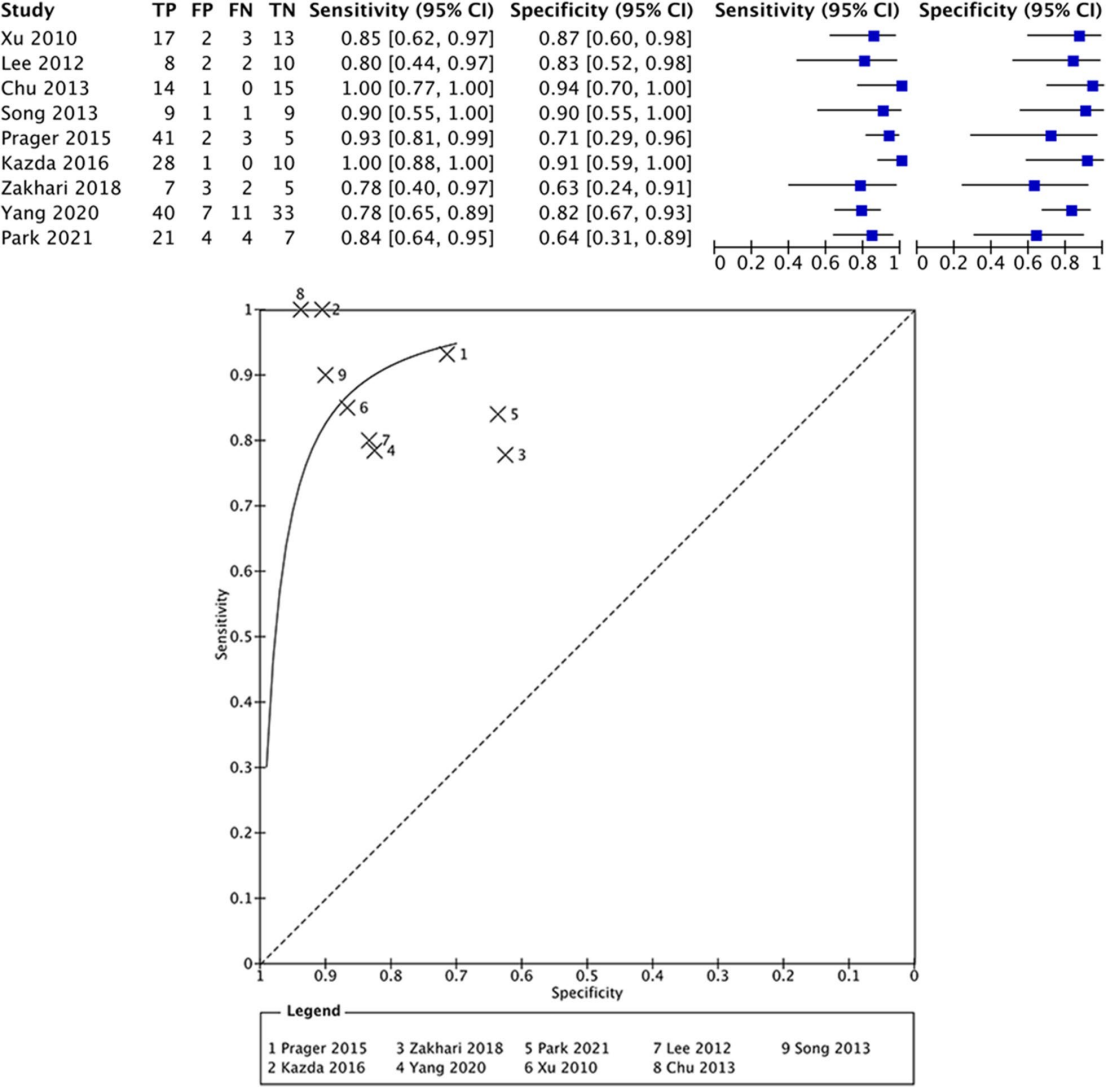
Summary receiver operator characteristics plot of sensitivity and 1-specificity ratios of the included studies

In total, 413 patients (252 males; 161 females) with an estimated mean age of 50.7 ± 1.3 years were included in this meta-analysis. All studies provided information with regard to the WHO grade of the glioma with the exception of the study of Zakhari et al. [21]. Seven patients suffered from a glioma WHO Grade 2, 101 from a glioma WHO Grade 3 and 288 from a glioma WHO grade 4. In all studies the diffusion tensor imaging related parameter, the *b*-values, concerned *b* = 0 s/mm^2^ and *b* = 1000 s/mm^2^. Only the study of Chu et al. (2013) also investigated an additional *b*-value of 3000 s/mm^2^ [22] (Table 1). ADC values were determined in different ways based on regions of interest (ROI) of enhancing lesions. In some papers, the ROI were manually drawn, some were manually placed circular ROI and Kazda et al. did not report how the region of interest was selected. On a meta-level, the mean ADC value of the patients in the TP group showed to be 1.13 × 10^−3^mm^2^/s with a 95%-confidence interval (95% CI) of 0.912 × 10^–3^–1.32 × 10^−3^mm^2^/s (*I*^2^ = 99%; *p* < 0.001). In the TRA group, on the other hand, the mean ADC value showed to be 1.38 × 10^−3^mm^2^/s with a 95% CI of 1.33 × 10^–3^–1.452 × 10^−3^mm^2^/s (*I*^2^ = 87%; *p* < 0.001). Meta-analysis showed that there was a mean difference of − 0.24 × 10^−3^mm^2^/s between the mean ADC metrics in TP and TRA. This indicated that there was a significant mean difference between ADC values in TP versus TRA (*p* = 0.005). However, a highly significant heterogeneity of the included studies was observed with an *I*^2^ of 87% (*p* < 0.0001) (Fig. 3). Figure 4 provides an overview of the sensitivity/specificity ratios of the included studies; only the paper of Zeng et al. (2007) provided insufficient data to be included in this part of the meta-analysis [15]. Pooled sensitivity showed to be 85% (95% CI 78.5–89.8%); pooled specificity showed to be 81% (95% CI 72.3–86.6%). Egger’s regression test showed no significant publication bias (*p* = 0.700).

**Table 1:** Overview of the included ADC studies and demographics of the included patients

Author	Total (*n*)	Mean age (SD/range)	Sex (M/F)	WHO grade (*n*)	*b*-values (s/mm^2^)	TP	Mean ADC value (SD)	95% CI	PsP	Mean ADC (SD);	95%-CI	TP	FP	FN	TN	Sensitivity/ Specificity (%/%)
						(*n*; lesions)	Enhancing lesion (× 10^–3^ mm^2^/s)		(*n*; lesions)							
										Enhancing lesion (× 10–3 mm^–2^/s)						
Zeng et al. [15]	55	43.67 (11.9) years	30/25	3 (36)	b0/1000	32	1.20 (0.80)	0.92–1.48	23	1.39 (0.90)	1.02–1.76	NR	NR	NR	NR	NR
				4 (19)												
Xu et al. [16]	35	45.2 (21–65) years	19/16	2 (4)	b0/1000	20	1.23 (0.20)	1.14–1.32	15	1.54 (0.17)	1.45–1.63	17	2	3	13	85/87
				3 (14)												
				4 (17)												
Lee et al. [17]	22	48.5 (15.8)	14-Aug	3 (3)	b0/1000	10	1.22 (0.39)	0.98–1.45	12	1.35 (0.17)	1.24–1.45	8	2	2	10	80/83
				4 (19)												
Chu et al. [22]	30	50.8 (25–72)	16/14	4 (30)	b0/1000/	15	1.27 (0.10)	1.07–1.46	15	1.33 (0.06)	1.30–1.36	14	1	0	15	100/94
					3000											
				12 unmethylated;												
				18 methylated												
Song et al. (2013)	20	57.1 (8.3); 44.4 (15.1)	10-Oct	4 (20)	b0/1000	10	1.27 (0.2)	1.18–1.44	10	1.44 (0.18)	1.36–1.52	9	1	1	9	90/90
				7 unmethylated; 13 methylated												
Prager et al. [19]	68	54.9 (22.6–79.4) years	51/17	3 (13)	b0/1000	58	1.38 (0.13)	1.35–1.41	10	1.59 (0.19)	1.47–1.71	41	2	3	5	93/71
				4 (55)												
				19 unmethylated; 12 methylated												
Kazda et al. [20]	39	51 (29–66)	28-Nov	4 (39)	b0/1000	29	0.61 (0.04)	0.59–0.63	10	1.33 (0.4)	1.08–1.58	28	1	0	10	100/91
Zakhari et al. [21]	17	55.9 (10.3) years	13-Apr	HGG	b0/1000	9	1.10 (0.21)	0.96–1.24	8	1.06 (0.27)	0.87–1.25	7	3	2	5	78/63
Yang et al. [11]	91	48.7 (14.2)(9–77) years	53/38	3 (29)	b0/1000	51	0.80 (0.08)	0.78–0.82	40	1.3 (0.05)	1.28–1.32	40	7	11	33	78/83
				4 (62)												
				22 IDHm; 69 IDHwt												
Park et al. [12]	36	52.3 (13.7)(*n* = 25) 57.1 (14.8)(*n* = 11)	18/18	2 (3)	b0/1000	25	1.28 (0.14)	1.23–1.33	11	1.47 (0.21)	1.35–1.59	21	4	4	7	84/64
				3 (6)												
				4 (27)

### Meta-analysis on the use of fractional anisotropy

**Fig. 5:**
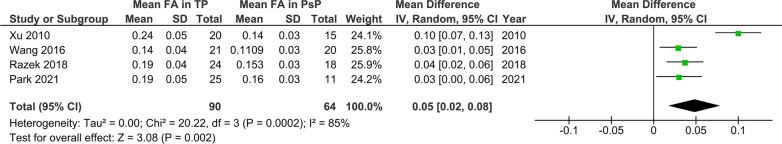
Forest plot of included studies on FA metrics

**Fig. 6:**
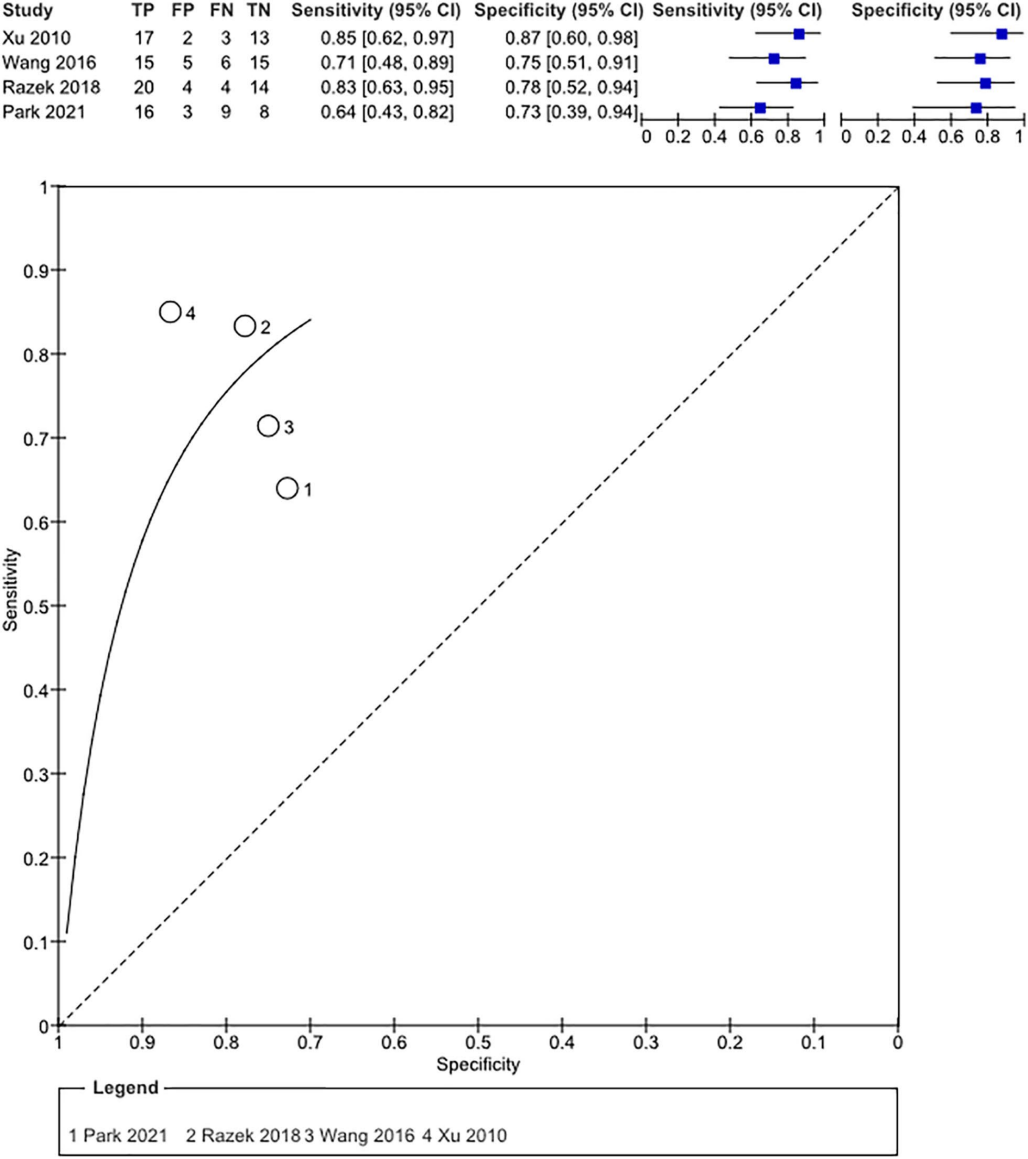
Summary receiver operator characteristics plot of sensitivity and 1-specificity ratios of the included studies

Four studies totaling 154 patients (85 males; 69 females; estimated mean age of 51.8 ± 4.6 years) were included in this analysis. Razek et al. [24] did not report mean age or WHO grade for individual patients, only that they included WHO 3 and WHO 4 gliomas. Studies that reported the individual participants’ characteristics showed that seven patients had WHO 2 gliomas, twenty had WHO 3 and 85 patients suffered from WHO 4 grade glioma. In total the studies found 90 TP and 64 TRA. In all studies, the b-values used were *b* = 0 s/mm^2^ and *b* = 1000 s/mm^2^. More information on baseline populations can be found in Table 2. All FA values are based on ROI in and around the contrast-enhancing lesion, in three studies they were manually drawn, Wang et al. used semi-automated segmentation. Mean FA value for TP was 0.19 (95% CI 0.189–0.194), while mean FA value for TRA was 0.14 (95% CI 0.137–0.143). The mean difference of FA values between TP and TRA groups was found to be 0.05 and showed to be significantly different (*p* = 0.002) (Fig. 5). Pooled sensitivity and pooled specificity showed to be 75.2% (95% CI 53.3–89.1%) and 77.6% (95% CI 60.5–90.4%), respectively (Fig. 6). Egger’s regression test again showed no significant publication bias (*p* = 0.200).

**Table 2:** Overview of the included FA studies and demographics of the included patients

Author	Total (*n*)	Mean age (SD/range)	Sex (M/F)	WHO grade (*n*)	*b*-values (s/mm^2^)	TP	Mean FA value (SD)	95% CI	TRA	Mean FA value (SD)	95%-CI	TP	FP	FN	TN	Sensitivity/ Specificity (%/%)
						(*n*; lesions)			(*n*; lesions)							
							Enhancing lesion			Enhancing lesion						
Xu et al. (2010)	35	45.2 (21–65)	19/16	2 (4)	b0/1000	20	0.24 (0.05)	0.218–0.262	15	0.14 (0.03)	0.125–0.155	17	2	3	13	85/87
				3 (14)												
				4 (17)												
Wang et al. (2016)	41	55.71 (11.83)	27/14	4 (41)	b0/1000	21	0.14 (0.04)	0.123–0.157	20	0.11 (0.03)	0.0969–0.123	15	5	6	15	71/75
Razek et al	42	NR	13-Nov	3 (NR)	b0/1000	24	0.20 (0.04)	0.184–0.216	18	0.15 (0.03)	0.136–0.164	20	4	4	14	83/78
				4 (NR)												
Park et al. (2021)	36	52.3 (13.7)(*n* = 25) 57.1 (14.8)(*n* = 11)	18/18	2 (3)	b0/1000	25	0.19 (0.05)	0.18–0.22	11	0.16 (0.03)	0.142–0.178	16	3	9	8	64/73
				3 (6)												
				4 (27)

## Discussion

Although of paramount importance in the follow-up and management of glioma patients, to date it is proven difficult to accurately differentiate between the TP and TRA. Because the outcome between TRA and TP is vitally different, early accurate differentiation of the two could help prevent re-intervention in TRA patients while also providing grounds for treatment in the TP patient group. This meta-analysis shows that, on a group level, the ADC and, to a lesser extent, FA values can be used to distinguish TP from TRA in post-treatment glioma patients (*p* = 0.005) with pooled sensitivities and specificities of 85% and 81%, and 75% and 78% for ADC and FA values, respectively. The estimated mean ADC and FA values of the patients in the TP group and in the TRA group were consistent with the theory that TP is the result of increased tumor cell proliferation, which causes a reduction in extracellular water diffusion, resulting in a lower ADC value [26, 27]. It has been hypothesized that decreased cellularity due to necrosis as a result of treatment damage (TRA) results in a lower FA value with more extracellular volume, whereas the FA value would increase due to an increased cellularity in TP when growing along existing white matter tracts [28]. The mechanisms driving TRA, however, remain partially elusive. It is believed that due to the (micro)vascular damage after radiation therapy, capillary leakage occurs, resulting in the production of cytotoxic and vasogenic edema [29]. In addition, oligodendroglial injury also plays an important role in the development of TRA [30]. Thereby, it is believed that TRA is reflected by relatively increased ADC value of the tissue [19], with a relative decrease of FA [9]. These hypotheses are corroborated by current findings.

The presented ADC data available for meta-analysis was limited when compared to the abundance of literature dealing with DWI in the follow-up of glioma. This is due to the use of different statistical measures and different outcomes in different papers. Research into different measures of the ADC value, like 5th percentile or relative ADC value could provide new information for the development of ADC maps [10, 27, 31]. However, the use of different measures might slow down further developments and future research due to limited availability of comparable data for meta-analysis. This would be disadvantageous as the ADC maps have also shown promising results with regard to identifying infiltrative patterns of glioma growth [32], predicting O^6^-methylguanine-DNA methyl-transferase (MGMT) methylation status [33] and predicting treatment outcomes and survival [33–35]. In addition, in patients who underwent laser interstitial thermal therapy for glioblastoma, the ADC value of the direct postoperative MRI scan (< 24 h) in the peritumoral region showed to be correlated with regions of later tumor recurrence [36].

The FA value is a scalar value between 0 and 1.0 and thereby is a more consistent metric between participants. Next to differentiating TP from TRA, the FA value has also been reported to be able to detect isocitrate dehydrogenase (IDH) status in oligodendroglial tumors to assess the prognosis and treatment options noninvasively and with an accuracy of about 80% [37]. Another possibility of FA value is to assess infiltration of tumor cells and predict sites of recurrence of glioma by analyzing peritumoral edema or by using distance-informed Track-weighted imaging [38, 39]. This shows promise for detecting invasion and aid in determining the clinical radiation target volume.

Additionally, there have been studies which add other metrics to the imaging diagnostics pipeline (multimodality MRI) in order to make the differentiation between recurrence and treatment-induced change more reliable. Perfusion magnetic resonance imaging (dynamic contrast enhancement, dynamic susceptibility contrast and arterial spin labeling) and magnetic resonance spectroscopy have been reported to be able to accurately distinguish between tumor tissue and radiation induced TRA [2, 20, 40]. PET-MR can be used to monitor treatment response in glioma and to detect recurrence [2]. It is important to note that all diagnostics have their own strengths and boundaries. Knowledge of the properties of these advanced imaging techniques can facilitate the synthesis of more evidence-based assessment of the tissue and help lead to accurate diagnosis of the problem at hand.

### Limitations and challenges for implementation of ADC and FA analysis in individual patients

A limitation of this review concerns the fact that the review was not registered in an international database of prospectively registered systematic reviews.

As shown in the present meta-analysis, the ADC and FA values can be used to distinguish TP and TRA in research settings on a group level. A prominent and characteristic limitation of DWI/DTI is the lack of validated diagnostic criteria on an individual level. This partly explains the different sensitivity and specificity values between research populations and further research is warranted in order to be able to specify and validate individual diagnostic criteria and improve the scope of use for DWI/DTI metrics.

Another general limitation for diffusion metrics is that the interpretation of values without context is highly ambiguous and the ADC/FA values are influenced by other factors such as clinical data (e.g., age, atrophy, other white matter defects), measurement purpose (i.e., detect IDH status or discriminate TRA from TP) as well as scanner type and protocol) which all can lead to significant intra-individual differences in ADC/FA values. Significant differences in ADC values can occur on an individual level depending on the scanner and scanning protocol used, thereby not only reflecting a difference between TP and TRA but also a difference on group level inflicted by scanner type. This is inherent to current standards for reporting diffusion weights with only *b*-values, rather than reporting the duration of, and time between, the pulsed magnetic field gradients encoding motion [41]. In order to tackle this shortcoming, we suggest standardizing acquisition or reporting these additional timings according to the FAIR-principles. Longitudinal single center studies using the same DWI protocol to follow, e.g., treatment response, do not experience this issue as long as they use their institutional reference values. Apart from only reporting *b*-values rather than DWI gradient timing, the low *b*-value used in current research (*b*0) does not take into consideration signal present from blood flow or perfusion which has not yet been attenuated. *b*-values above approximately 100 s/mm^2^ have been shown to attenuate these signals, and only signal from diffusing water remains[41]. Future studies might consider calculating DWI metrics using *b*-values of 100 s/mm^2^ as the lower value in order to avoid perfusion and blood flow effects to assess possible differences in diagnostic outcome and reliability.

It should also be taken into consideration that, despite the general recommendation that the radiological follow-up of glioma should be performed by experienced radiologists using with a multiparametric MRI protocol containing up-to-date sequences. Using FA parameters in the follow-up of glioma implies that all MRI scanners have access to this modality. This is, however, not always the case, as the value of the DTI sequences is still being investigated. Diffusion-weighted imaging, on the other hand, is available as a standard sequence and therefore widely available for the follow-up of glioma. Therefore, it is recommended to focus future research on harmonization of imaging protocols and diffusion metrics in order to create more interchangeable ADC values. This will allow for more reliable diagnoses based on diffusion metrics.

The different means of tissue mapping (ROI/manual segmentation) are another variable thwarting normalization of the process. Advanced analysis is warranted in order to determine the best course of action for tissue mapping. Furthermore, the co-occurrence of TP and necrotic changes [42, 43] impact the diffusion values which further complicates usage and standardized application in the daily clinical setting. It is also important to note that Xu et al. and Park et al. included WHO 2 glioma patients, which are defined as low-grade gliomas and could respond differently to treatment and possibly influence imaging results, as this paper focuses on high-grade gliomas.

Additionally, the quality of the majority of the papers on this subject was insufficient as most authors did not report standard metrics (e.g., mean, standard deviation, 95% CI) though instead reported values which produced significant results in relatively small datasets. Future research should focus on the publication of study data following the FAIR principles (i.e., findable, accessible, interoperable, re-usable). The FAIR principles underline the importance of the capacity of computational systems to find, access, interoperate, and reuse data with none or minimal human intervention as the data which has to be dealt with shows an increase in volume and complexity and is created significantly faster than before [44].

Finally, the clinical implementation of the reported results of this meta-analysis will be hampered by inter-subject variability of the ADC/FA maps. Therefore, an externally validated prediction model with diagnostic criteria on an individual level is necessary and cannot be derived from group analyses. This could be achieved by using a uniform method of normalization on a prospective cohort, for example by obtaining the ADC value from the same region in a standardized atlas, using a normalized scanning protocol with a standardized method of tissue mapping.

## Conclusion

The current meta-analysis showed that ADC and FA metrics can accurately distinguish TP and TRA in groups of patients. For further clinical implementation, harmonization of imaging protocols and reading procedures is required. In addition, well-validated criteria that can be used in individual patients, based on clinical studies, preferably in large, prospective cohorts with FAIR publication of study data, are warranted for this field to further develop.

## Data Availability

The datasets generated during and/or analyzed during the current study are available from the corresponding author at reasonable request.

## Abbreviations

ADC: Apparent diffusion coefficient
DTI: Diffusion tensor imaging
DWI: Diffusion-weighted imaging
FA: Fractional anisotropy
FAIR: Findable, accessible, interoperable, reusable
IDH: Isocitrate dehydrogenase
MGMT: O^6^-methylguanine-DNA methyltransferase
PRISMA: Preferred reporting items for systematic reviews and meta-analyses
PWI: Perfusion-weighted imaging
RANO: Response Assessment in Neuro-Oncology
ROI: Region of interest
TP: Tumor progression
TRA: Treatment-related abnormalities
VEGF: Vascular endothelial growth factor
WHO: World Health Organization
